# Long-term patient reported outcomes and quality of life after syndactyly separation

**DOI:** 10.1177/17531934251380997

**Published:** 2025-10-22

**Authors:** Noora Nietosvaara, Niko Kämppä, Yrjänä Nietosvaara, Petra Grahn

**Affiliations:** 1 Department of Orthopedics and Traumatology, Helsinki University Hospital, University of Eastern Finland, Finland; 2 Department of Hand Surgery, Helsinki University Hospital and University of Helsinki, Finland; 3 Department of Paediatric Orthopedics and Traumatology, New Children’s Hospital, Helsinki University Hospital, University of Helsinki, Finland; 4 Department of Paediatric Surgery, Kuopio University Hospital, Finland

**Keywords:** Congenital hand differences, long-term outcomes, patient-reported outcomes, quality of life, syndactyly, syndactyly reconstruction

## Abstract

**Introduction:**

We assessed the long-term quality of life, cosmesis and pain after childhood syndactyly separation.

**Methods:**

A retrospective review identified 155 patients (⩾10-year follow-up) of whom 76 participated with a median follow-up period of 18.2 years (IQR 12.4 to 38.3). Patient-reported outcomes were assessed using the Quick Disabilities of the Arm, Shoulder, and Hand questionnaire, EuroQol-5 Dimension-5 Level, and visual analogue scales (0–10) for function, cosmesis and pain.

**Results:**

Uncomplicated cases had significantly better patient reported outcomes (0 vs. 11.4 and 1.0 vs. 0.89; *p* < 0.01), and higher functional scores (9.0 vs. 7.0, *p* < 0.05) and cosmetic scores (8.2 vs. 7.0, *p* > 0.05). Complicated cases presented with more cold intolerance (58 vs. 14%, *p* < 0.01). Pain scores were similar in both groups, with 16–18% reporting some pain in the past week.

**Conclusion:**

Syndactyly reconstruction yields satisfactory subjective long-term outcomes. Complicated cases had poorer function and more cold sensitivity but remained overall favorable.

**Level of evidence:**

IV

## Introduction

With a reported incidence of 2 per 10,000 live births, syndactyly is one of the most common upper limb differences (Braun et al., 2016; Ekblom et al., 2010; Kozin and Zlotolow, 2015). Syndactyly separation is performed for both cosmetic and functional reasons, thus making it one of the most common surgical procedures for congenital upper limb differences (Ekblom et al., 2010; Giele et al., 2001; Koskimies et al., 2011; Koskimies-Virta et al., 2020). Surgery is typically performed from around 1 year of age, depending on the severity of the deformity (Koskimies-Virta et al., 2020). However, surgery performed too early can lead to late complications, such as web creep or contractures, which may cause deformities as the hand develops (Dao et al., 2004). Traditionally, syndactyly separation has been performed with zigzag incisions and full-thickness skin grafts. However, good early results have also been reported with different graftless techniques (Grahn et al., 2020; Kozin and Zlotolow, 2015) and the use of skin substitute (Bohn et al., 2025; Landi et al., 2014).

Despite the prevalence of syndactyly and the frequent performance of syndactyly separation surgery, limited data exists on the long-term quality of life (QoL) of these patients, particularly regarding cosmesis and functional outcomes, as well as pain. This study aims to answer these questions by evaluating the QoL in adolescents and adults who underwent syndactyly separation during early childhood, using validated patient-reported outcome measures (PROMs).

## Methods

This was a retrospective long-term follow-up study conducted at New Children’s hospital, Helsinki University hospital. Patients who had undergone syndactyly separation were identified by searching the hospital records using the relevant diagnostic codes from the International Classification of Diseases versions 8–10. Institutional and ethical review board approval was obtained (approval number 564/2024). Informed consent was obtained from all participants. For those under 16 years of age, consent was also obtained from a parent or guardian.

### Patient selection and grouping

Patients were included if a minimum of 10 years had elapsed from their last syndactyly surgery. To ensure an independent evaluation of outcomes, the follow-up assessments were conducted by two surgeons (NK and NN), who were not involved in the treatment of the participants.

Participants were divided into two groups for analysis:

1. Uncomplicated syndactyly (*n* = 49, 71 hands) – simple incomplete, simple complete, or complex syndactyly.
2. Complicated syndactyly (*n* = 27, 38 hands).

**Figure 1.**
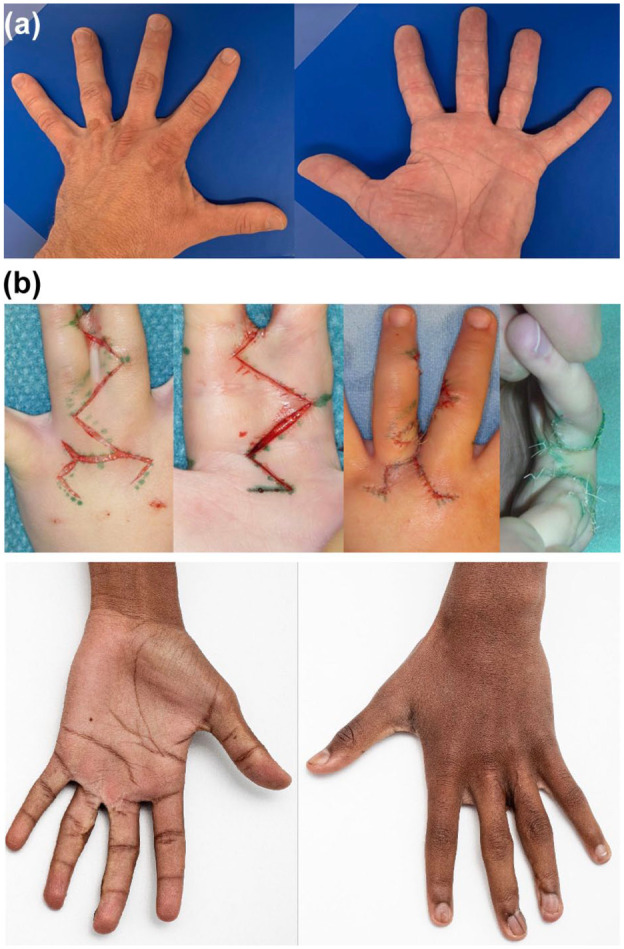
(A) Syndactyly separation during 1965 and 2002 was usually performed with the method described by Cronin (1956) or its modification and inguinal full-thickness skin grafts – outcome of third web space simple complete syndactyly separation 47 years after surgery. (B) Our standard technique to separate simple and complex syndactyly between 2003 and 2011 was a graftless technique using zigzag incisions and a dorsal hexagonal flap – outcome of third web space complex complete syndactyly separation 15 years after surgery.

Simple syndactyly separation between 2003 and 2011 utilized the graftless technique: zigzag incisions and defatting combined with a hexagonal dorsal flap to reconstruct the web (Figure 1). Inguinal full thickness skin grafts were used in the separation of complicated syndactyly. Surgery was performed by paediatric surgeons between 1965 and 1999, and from 2000 to 2011, by YN and other paediatric orthopedic surgeons trained by him. The treating surgeons’ level of expertise ranged from 2 to 5 (Tang and Giddins, 2016).

### Assessment

Participants completed the Quick Disabilities of the Arm, Shoulder, and Hand (QDASH) questionnaire, EuroQol-5 Dimension-5 Level (EQ-5D-5L) and a visual analog scale (VAS) for function, cosmesis, and pain. In contrast, VAS responses for cosmesis and function were collected separately for each affected hand, enabling side-specific analysis in patients with bilateral syndactyly.

#### QDASH

This 11-item questionnaire, with optional modules for work and sports/performing arts activities, assesses disability and symptoms in the upper extremity. Scores range from 0 to 100, with higher scores indicating greater disability (Kennedy et al., 2011). Although not validated for congenital hand deformities, QDASH is widely used in other upper-extremity contexts (Wormald et al., 2019). The minimal clinically important difference is reported as 8.0 to 18.1, with a pooled value of 11.97 in a recent meta-analysis (Galardini et al., 2024).

#### EQ-5D-5L

This is a generic PROM that assesses health-related QoL across five dimensions: mobility, self-care, usual activities, pain/discomfort, and anxiety/depression. Responses are recorded on a five-level Likert scale and summarized into an index score ranging from 0 (death equivalent) to 1 (full health). The English value set was used owing to the lack of a Finnish version (Devlin et al., 2018).

#### VAS for function and cosmesis

Patients rated function and cosmesis on a 0 to 10 scale, with higher scores indicating better outcomes.

#### VAS for pain

Pain during the previous week as well as current pain was assessed by patients on a 0–10 scale, with higher scores indicating more pain.

### Statistics

Continuous variables were assessed for normality using quintile–quintile plots and the Shapiro–Wilk test, both of which indicated non-normal distributions. As a result, medians with interquartile ranges (IQRs) or 95% confidence intervals (95% CIs) were used to summarize continuous data. Categorical variables were presented as counts (*n*) and percentages of the study population or group total. For groupwise comparisons, the non-parametric Mann–Whitney *U*-test was used for continuous variables, and the chi-square test was applied for categorical variables. A non-parametric basic-type bootstrapping method was employed to estimate 95% CIs.

A multiple linear regression model was used to further assess relationships between PROMs and syndactyly. Adjustment for sex and range of motion abnormality was done for all models. As bilateral affliction is not accounted for in QDASH or EQ-5D-5L, but VAS data were collected by side, bilateral affliction was only added to the models assessing QDASH and EQ-5D-5L data. A *p-*value < 0.05 was considered statistically significant.

## Results

A total of 155 patients met the inclusion criteria based on hospital records between 1965 and 2011. Current contact information was available for 90 patients, all of whom were invited to a clinic follow-up appointment. Of these, 76 patients (122 webs) agreed to participate in the study. Syndactyly separation was usually performed with the Cronin method (1956) or its modification (Figure 1) in the 36 children operated on during 1965–2002, but the exact surgical technique could not be confirmed in 24 patients who had either incomplete (*n* = 11) or missing (*n*=13) operative notes. The mean age of participants at final surgery was 3.3 years (range 0.3–13.3) and at follow-up 29 years (range 12–64) (Table 1). All patients included in the study except two (Black) were Caucasian and had reached adult hand size.

**Table 1.** Demographic and clinical characteristics of participants.

	Uncomplicated	Complicated	Total	Simple vs. complicated
	(*n* = 49)	(*n* = 27)	(*n* = 76)	*p*
Sex				0.21
Female	10 (20%)	9 (33%)	19 (25%)	
Male	39 (80%)	18 (67%)	57 (75%)	
Age at follow-up (years)				
Median (IQR)	27.6 (15.5–41.0)	17.5 (13.8–35.9)	22.7 (14.8–40.9)	0.87
Follow-up time (years)				
Median (IQR)	23.5 (13.4–38.5)	15.4 (11.5–31.8)	18.2 (12.4–38.3)	
Handedness				0.41
Right	38 (78%)	23 (82%)	61 (79%)	
Left	7 (14%)	3 (11%)	10 (13%)	
Ambidextrous	3 (6%)	0 (0%)	3 (4%)	
Data missing	1 (2.0%)	2 (7.1%)	3 (3.9%)	
Dominant hand affected				0.49
No	19 (39%)	12 (44%)	31 (41%)	
Yes	29 (59%)	13 (48%)	42 (55%)	
Data missing	1 (2.0%)	2 (7.4%)	3 (3.9%)	
Affected limb				0.87
Right	8 (16%)	5 (19%)	13 (17%)	
Left	18 (37%)	11 (41%)	29 (38%)	
Bilateral	23 (47%)	11 (41%)	34 (45%)	
Other anomalies				0.95
No	17 (35%)	9 (33%)	26 (34%)	
Yes	31 (63%)	17 (63%)	48 (63%)	
Data missing	1 (2.0%)	1 (3.7%)	2 (2.6%)	
Relatives with syndactyly				0.04
No	31 (63%)	22 (81%)	53 (70%)	
Yes	16 (33%)	3 (11%)	19 (25%)	
Data missing	2 (4.1%)	2 (7.4%)	4 (5.3%)	

* The uncomplicated syndactyly group includes simple incomplete, simple complete and complex syndactyly cases.

All 76 patients answered the main QDASH questionnaire as well as the VAS for current and worst pain. The QDASH work module was answered by 42 patients (*n* = 31 uncomplicated, *n* = 11 complicated), while the sports and performing arts module was answered by by 59 patients (*n* = 39 uncomplicated, *n* = 20 complicated). One person with complicated and one with uncomplicated syndactyly did not finish the EQ-5D-5L questionnaire. Cosmetic and function VAS data were not retrieved from one in the uncomplicated and two participants in the complicated group.

### PROM outcomes

**Figure 2.**
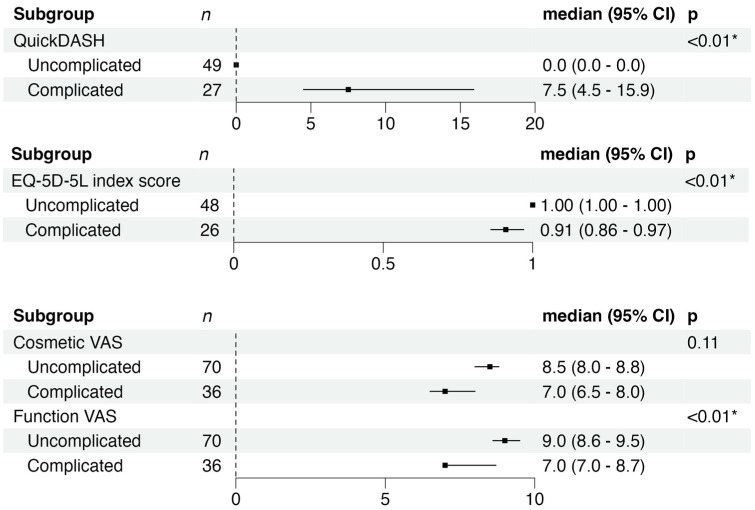
Forest plots showing median scores and 95% confidence intervals (CI) for the Quick Disabilities of the Arm, Shoulder, and Hand (QDASH) questionnaire, cosmetic visual analog scale (VAS), functional VAS, and EuroQol-5 Dimension-5 Level (EQ-5D-5L) index scores in uncomplicated and complicated syndactyly groups. Statistically significant differences were observed for QDASH, functional VAS and EQ-5D-5L (*p* < 0.01), while differences in cosmetic VAS scores were not significant (*p* = 0.11). QDASH and EQ-5D-5L were scored once per participant, whereas VAS scores for cosmesis and function were collected per affected hand. *n* = number of responses.

For the entire cohort, the median QDASH score was 2.3 (95% CI 0 to 4.5), with significantly better scores for patients with uncomplicated syndactyly compared with those with complicated syndactyly (Figure 2). Work, sports and performing arts analysis revealed no significant differences between groups, with both scoring 0 (95% CI 0 to 0) for the work module and 0 (95% CI 0 to 0 vs. 0 to 6.25) for the sports and performing arts module (*p* > 0.05 for both). The EQ-5D-5L index score was also significantly higher in the uncomplicated group (Figure 2).

Functional VAS scores were significantly higher in the uncomplicated group compared with the complicated group, indicating better perceived functionality. Cosmetic VAS scores were higher in the uncomplicated group but this difference did not reach statistical significance (*p* > 0.05) (Figure 2).

Current and worst pain scores were uniformly low across all participants, with a median of 0 (95% CI 0 to 0) for both groups. Despite this, some pain in the previous week was reported in 19 out of 109 affected hands, with similar proportions in the uncomplicated (13 of 71, 18%) and complicated (6 of 38, 16%) groups with no statistical significance difference between groups. Cold sensitivity was more common in the complicated group (12 of 27, 44% vs. 8 of 47 (data missing for two participants in uncomplicated group, 17%, *p* = 0.01).

Multiple linear regression models confirmed significant differences between uncomplicated and complicated syndactyly (Table 2). Complicated syndactyly was associated with higher QDASH scores, lower EQ-5D-5L index scores, and lower functional VAS scores.

**Table 2.** Differences in QuickDASH, EQ-5D-5L and function VAS between uncomplicated and complicated syndactyly using multiple linear regression with uncomplicated syndactyly as reference and adjustment for bilateral affliction and abnormal AROM.

PROM	Estimate (*β*)	95% CI	Standard error	*p*-Value
QuickDASH	7.97	3.62–12.32	2.22	<0.01***
EQ-5D-5L	−0.05	−0.09 to −0.01	0.02	0.01*
Functional VAS	−0.97	−1.91 to −0.03	0.47	0.04*

* *p* < 0.05; **p < 0.01; ***p < 0.001.

## Discussion

In this study, we evaluated the long-term QoL in adolescents and adults who underwent syndactyly separation during early childhood, using validated patient-reported outcome measures.

Previous publications on syndactyly have mainly reported on short-term results of different surgical techniques based on physician-reported objective outcome measures (Barabás and Pickford, 2014; Dong and Wang, 2017; Landi et al., 2014; Mallet et al., 2013; Mericli et al., 2015; Sharma et al., 2009; Sherif, 1998; Tian et al., 2017; Wang et al., 2020; Yoon and Jones, 2019). There remains a paucity of PROMs and longer-term QoL reports; the small number of studies on long-term outcomes are limited by small numbers the lack of validated PROMs (Lumenta et al., 2010; Widerberg et al., 2016).

Lumenta et al. (2010) reported good functional long-term outcomes of simple syndactyly release (zigzag incisions with skin grafts with palmar flaps for web reconstruction) in 19 patients after a mean follow-up time of 11.5 years. Widerberg et al. (2016) reported good functional and cosmetic outcomes (median QDASH scores 2.3, range 0–11.4) and minimal effect on choice of occupation and leisure activities in 19 patients after separation of simple syndactyly (zigzag incisions without skin grafts with a dorsal flap for web reconstruction) after a follow-up of 10–24 years. According to an online survey most aspects of health-related QoL were comparable with those of the general population in 2- to-18-year-old patients with simple (*n* = 14) and complex (*n* = 37) syndactyly 1–9 years after surgery (Park et al., 2020). The response rate was 41% (51 of 124) and surveys were mostly answered by parents (42 of 51). In our earlier study, parents of children with an upper limb difference rated the appearance of congenitally different hands higher compared with other respondents (Nietosvaara et al., 2021).

Our study confirms these earlier findings with longer-term data on a larger number of patients. Among 76 patients with 10–59 years of follow-up, those treated for uncomplicated syndactyly reported no functional limitations in daily life and perfect health utility scores (EQ-5D-5L index = 1.0), indicating full health across all assessed domains. Patients treated for complicated syndactyly also reported high EQ-5D-5L index scores (mean index = 0.9), although they more frequently described subjective limitations in hand function. Notably, these limitations did not interfere with work, sports participation or performance-related activities, suggesting a minimal impact on overall QoL. These results align with findings in other congenital upper limb conditions. Markiewicz et al. (2023) studied 127 adults with thalidomide-related upper limb differences and found a median EQ-5D-5L index of 0.6, indicating moderate impairment, and a strong correlation between upper limb disability and declining QoL. Importantly, 70% of patients reported worsening health-related QoL with age, highlighting the potential cumulative burden of upper limb anomalies over time. Their study reinforces the importance of collecting long-term patient-reported outcomes to understand the evolving impact of congenital upper limb conditions into adulthood.

The cosmetic appearance of simple complete syndactyly of the third web (mean score 2.89 out of 5, SD 1.05) was rated significantly lower than that of a normal hand (mean score 4.43 out of 5, SD 0.85) in Finland, Austria and Singapore by laypersons (Nietosvaara et al., 2021). Syndactyly separation has been shown to be effective in enhancing hand appearance: in our recent study, 98% of 1165 lay respondents found postoperative images of simple complete syndactyly more aesthetically pleasing than preoperative ones (Nietosvaara et al., 2023). In a separate study by Widerberg et al. (2016), the mean cosmetic VAS score following traditional zigzag incisions with skin grafts (using various and often undocumented web reconstruction techniques) was 79 (range 38–100). In that cohort, patients with uncomplicated syndactyly reported a mean score of 8.5, and those with complicated syndactyly a mean score of 7.0. Although these values reflect generally favorable aesthetic outcomes, the lowest reported scores, 5.0 and 6.5, respectively—suggest that cosmetic satisfaction varies and that further improvements in technique may be warranted to achieve more consistently high aesthetic outcomes.

To the best of our knowledge there are no previous studies assessing pain after finger syndactyly release. Both the QDASH questionnaire and the EQ-5D-5L index score address pain; QDASH specifically addresses pain during the last week. Every sixth patient in our study reported pain. The specific reason for pain was not investigated in our study. Pain could be related to multiple issues, e.g. tight scars limiting finger motion or possible iatrogenic digital nerve injuries following release surgery. Given the long follow-up time for some participants, pain could also be related to issues, such as arthritis, that are not related to the syndactyly surgery.

Widerberg et al. (2016) showed that 37% of simple syndactyly patients reported some degree of cold sensitivity using a validated cold intolerance scale, but none exceeded scores that were considered abnormal. Their study found twice as much cold sensitivity compared with our report of 17% in uncomplicated cases; this might have to do with the use of the Cold Intolerance Symptom Severity questionnaire, which involves a more detailed questioning than our single yes/no cold sensitivity question. Cold sensitivity was reported in almost half of cases with complicated syndactyly in our study. More extensive surgery runs a higher risk for direct digital nerve injuries and extensive scarring, which are possible explanations for the high prevalence of cold intolerance in patients with complicated syndactyly.

The strengths of this study include the relatively large patient cohort with a median follow-up time of 18 years. The follow-up rate of 49% is moderate, but it can be considered satisfactory considering the 50-year-long time frame of the study. The use of validated PROMs improves the reliability of the results. The inclusion of complicated cases can be considered valuable because a subgroup analysis could be made. However, there are several limitations; owing to its retrospective design there is a potential recall bias. Secondly, the lack of a control group prevents comparisons with untreated cases, and subjective measures like VAS for cosmesis and pain may reflect individual biases. Thirdly, missing or incomprehensive surgical reports in some older cases was a drawback. Fourthly, the single-centre setting may also limit generalizability, and the use of EQ-5D-5L values from another country may slightly impact cultural relevance of our results. Finally, while PROMs provide valuable insight into the subjective outcome, objective long-term outcomes should also be reported. Therefore, we are currently in the process of assessing scar quality, finger sensation, alignment and range of motion of these patients.

This study highlights long-term patient-reported outcomes following childhood syndactyly surgery using traditional techniques. While most patients reported high satisfaction and QoL, pain and cold intolerance remained common, especially after complicated cases. These findings emphasize the value of validated PROMs and support more informed counselling on long-term expectations. Future research should include prospective multicentre studies incorporating qualitative feedback from patients to enhance generalizability across diverse cultural and healthcare settings. The reason for cold sensitivity is unclear and it should be investigated to develop protective measures.
